# Sonographic normative range of uterine and ovarian dimensions and their correlation with anthropometric and hormonal parameters among Indian women of reproductive age (18–40) years

**DOI:** 10.3389/fmed.2026.1765600

**Published:** 2026-07-16

**Authors:** P. K. Jabbar, Jayasree L., Abilash Nair, Sreejith Babu, Poulami Mazumder, Taruna Arora, Remya Nair, Jayakumari C., Khalid Ul Islam Rather, Imtiyaz Ahmad Wani, Nirmala C., Anuja Elizabeth George, Neena Malhotra, Devasenathipathy Kandasamy, Sarita Agrawal, Roya Rozati, Beena Joshi, Rakesh Sahay, Amlin Shukla, Subhankar Chowdhury, Vanita Suri, Prasanta Kumar Bhattacharya, Mohammad Ashraf Ganie

**Affiliations:** 1 Government Medical College, Thiruvananthapuram, India; 2 Division of Reproductive, Child Health and Nutrition, Indian Council of Medical Research, New Delhi, India; 3 Department of Endocrinology, Sher-i-Kashmir Institute of Medical Sciences, Srinagar, India; 4 Department of Obstetrics and Gynaecology, Sree Mookambika Medical College, Kulasekharapuram, India; 5 Department of Dermatology and Venereology, Government Medical College, Thiruvananthapuram, India; 6 Department of Obstetrics and Gynaecology, All India Institute of Medical Sciences, New Delhi, India; 7 Department of Radiodiagnosis, All India Institute of Medical Sciences, New Delhi, India; 8 Department of Obstetrics and Gynaecology, All India Institute of Medical Sciences, Raipur, India; 9 Department of Obstetrics and Gynaecology, Maternal Health and Research Trust, Hyderabad, India; 10 Department of Operational and Implementation Research, National Institute for Research in Reproductive and Child Health, Mumbai, India; 11 Department of Endocrinology, Osmania Medical College, Hyderabad, India; 12 Department of Endocrinology and Metabolism, Institute of Post-Graduate Medical Education and Research, Kolkata, India; 13 Department of Obstetrics and Gynaecology, Postgraduate Institute of Medical Education and Research, Chandigarh, India; 14 Department of General Medicine, North-Eastern Indira Gandhi Regional Institute of Health and Medical Sciences, Shillong, India

**Keywords:** hormonal profile, India, normative values, ovarian dimensions, reproductive-aged women, ultrasound, uterine dimensions

## Abstract

**Background:**

The Indian population exhibits distinct anthropometric characteristics compared to western populations, necessitating the establishment of region-specific normative values for uterine and ovarian dimensions.

**Objective:**

This study aimed to derive normative reference ranges for ovarian volume, follicle number, uterine size, and endometrial thickness among Indian women of reproductive age and its correlation with anthropometric and hormonal parameters.

**Methodology:**

A cross-sectional, community-based study was conducted among 1648 Indian women aged 18–40 years from diverse geographic regions. Participants were selected using multistage sampling. Pregnant or lactating women and women with known gynecological disorders were excluded. Clinical, biochemical, hormonal and radiological assessments were obtained using standardized protocols.

**Results:**

The mean uterine length measured via transabdominal ultrasound was 7.09 ± 1.64 cm, and transvaginal ultrasound showed a length of 7.11 ± 1.83 cm. Ovarian volumes were 7.06 ± 3.44 cc (left) and 7.26 ± 3.29 cc (right) using transabdominal scans, while transvaginal scans showed 7.12 ± 3.38 cc (left) and 7.45 ± 3.28 cc (right). The mean follicle count was 6.05 ± 3.18 (left ovary) and 5.89 ± 3.03 (right ovary) via transabdominal ultrasound. Ovarian volume was negatively correlated with age, while positively correlated with body weight, height, hirsutism score, insulin resistance, and testosterone. Endometrial thickness correlated positively with age and waist circumference but negatively with body weight, BMI, skin fold and triceps thickness.

**Conclusion:**

This study provides comprehensive normative ultrasound ranges for dimensions of uterus and ovaries in Indian women of reproductive age and its correlation with anthropometric and hormonal parameters. These normative values will aid in better diagnosis and management of reproductive health disorders in the Indian population.

## 1 Introduction

Indians constitute one sixth of the world’s population. It is a vast country with a diverse population, and this diversity is reflected in the genetic variation observed across different regions. A variety of ancestral components, social stratification, endogamy, and intricate admixture patterns all contribute to the complexity of this genetic variation (1).

This results in differences in the anthropometric measurements of Indian population as compared to the western population (2, 3). For assessing reproductive health and detecting gynecological problems, accurate evaluation of uterine and ovarian morphology is essential. Accurate assessment of uterine and ovarian morphology is necessary for evaluation of reproductive health and diagnosing gynecological disorders. Hence, it is essential to establish reference values of different reproductive organ sizes specific to Indian population. Ultrasound derived uterine and ovarian parameters are required for the diagnosis of various gynecological disorders, pediatric disorders, including precocious puberty, ambiguous genitalia, and in the investigation of abdominal and pelvic masses (3–5).

These parameters are often included in formulation of disease guidelines (5). By measuring the normal uterine dimensions, and correlating it with clinical conditions it aides in diagnosis of pelvic organ pathologies. The measurement of ovarian dimension is done routinely for diagnosis and treatment of a number of reproductive conditions. In women of reproductive age, the size and volume of the ovary serve as an indirect measure of the ovarian reserve (2). Ovarian volume serves as diagnostic criteria for polycystic ovary syndrome and may be of value in screening for ovarian cancer (6, 7).

It has been observed that girls with precocious puberty have significantly increased ovarian volumes as compare to normal population (3). Similarly, ovarian follicle number and ovarian antral follicle count (AFC) are helpful markers of infertility and assist in evaluation of reproductive health, for predicting the risk of menopause and for the diagnosis of hyperandrogenic anovulation (8).

Endometrial thickness is an indicator of hormonal action and a useful parameter to detect endometrial carcinoma (9). The USG derived parameters of ovarian volume, follicle number, uterine size and endometrial thickness vary with race and genetics. Majority of normative data for reproductive organ dimensions have been derived from western populations and due to unique ethnic, genetic, dietary, and lifestyle that may not be directly applicable to Indian population. There are limited numbers of studies done on these parameters in Indian population. Few studies which are done have limited sample size. This study was done to derive the normal reference range of ovarian volume, follicle number, uterine size, and endometrial thickness by including Indian women from various geographic regions of India.

## 2 Materials and methods

### 2.1 Study design

A community-based, cross-sectional study was conducted which included women belonging to the age group of 18–40 years from both rural and urban areas of 9 sites from Five zones (North, South, East, Northeast and Central) of India.

### 2.2 Sampling method

Participants were selected through a multistage sampling method, incorporating both urban and rural regions, with polling booths serving as the primary sampling units as described in previous study (10). The coordinating center used a random selection procedure to choose these booths. From each selected booth, the first 50 women listed in the electoral roll who met the study’s inclusion and exclusion criteria were enrolled from 9 different sites.

### 2.3 Ethical consideration

Prior to initiating any study-related procedures, written informed consent was secured from each participant ensuring they understand the study objectives, their responsibilities, and any potential implications of their involvement.

Ethical approval for this study was obtained from Institutional Ethics Committees at each of the 9 research locations which includes Human Ethics Committee of Government Medical College, Trivandrum; Institutional Ethics Committee of Sher-i-Kashmir Institute of Medical Sciences, Srinagar; Institutional Ethics Committee of Postgraduate Institute of Medical Education and Research, Chandigarh; Institutional Ethics Committee of All India Institute of Medical Sciences, Raipur; Institutional Ethics Committee of All India Institute of Medical Sciences, Delhi; Institutional Ethics Committee of Institute of Post Graduate Medical Education and Research, Kolkata; Institutional Ethics Committee of North Eastern Indira Gandhi Regional Institute of Health and Medical Sciences, Shillong; Institutional Ethics Committee of Osmania Medical College, Hyderabad; and Institutional Ethics Committee of Mahavir Hospital and Research Center, Hyderabad;. All methods were performed in accordance with the relevant guidelines and regulations of these committees.

### 2.4 Inclusion and exclusion criteria

(i) Inclusion Criteria: Indian healthy parous and nulliparous women aged 18–40 years from diverse geographic regions across 9 sites with no known clinical or biochemical evidence of any disease or pathology.

(ii) Exclusion Criteria:

1. Pregnant or lactating women,
2. Individuals with cognitive or physical impairments that hindered their ability to complete the questionnaire,
3. Women using medications such as steroids, androgens, oral contraceptives, antiepileptic drugs, or any agents known to affect glucose or lipid metabolism,
4. And woman with a clinical, biochemical, or radiological diagnosis of polycystic ovary syndrome (PCOS) or a history of pelvic disease were not included in the study.

### 2.5 Sample size

Sample size calculated using the formula, *N* = 4Zα^2^S^2^/W^2^, where S is the standard deviation and W is the desired total width of confidence interval, taken as 0.4. The derived sample size was 624.

Although the minimum required sample size was calculated as 624, a total of 1648 eligible participants were available during the study period. Therefore, all eligible cases were included to enhance the statistical power and ensure better population representativeness. Utilizing the full dataset (*n* = 1648) also improved the precision of estimates, reduced the margin of error, and enabled more reliable subgroup analyses, thereby strengthening the overall validity of the study findings.

(i) Uterine size-based sample size:

From the study by Chen et al. (11) S being 2.55 and W being 0.4 for uterine size. The derived sample size was 624.

(ii) Ovarian volume-based sample size:

From Danjem et al. study (12) W being 0.4 and S being 2.16 for ovarian volume, sample size was calculated to be 448.

### 2.6 Data collection

Structured predesigned questionnaire was used for capturing demographic and clinical information. The screening questionnaire was divided into three sections:

(1) Personal details,
(2) Menstrual history and clinical information, and
(3) History of any systemic diseases.

Other Investigations:

(4) Clinical Assessment (done between the cycle’s second and seventh days)
(5) Biochemical Assessment (done between the cycle’s second and seventh days)
(6) Hormonal Assessment (done between the cycle’s second and seventh days)
(7) Radiological Assessment (done during follicular phase of their menstrual cycle)

After obtaining written consent from participants the data was collected, entered in MS Excel, and analyzed using SPSS version 16.

#### 2.6.1 Clinical investigation

Clinical investigation included anthropometric assessment, hirsutism scoring, evaluation of acne, oligomenorrhea, androgenic alopecia and primary infertility.

#### 2.6.2 Anthropometric assessment

Study personnel’s were specially trained for the study performed anthropometric assessments, including measurements of height, weight, waist circumference, skinfold thickness on the dorsum of the hand and triceps, blood pressure, and a thorough systemic examination.

Height and Weight: Standard equipment was used to measure the subject’s height and weight while they were wearing light clothing and no shoes. A digital scale that had been calibrated was used to measure weight to the closest 0.1 kg. Using a stadiometer and conventional procedure, height was measured to the closest 0.1 cm.

Waist and Hip Circumference: Using a measuring tape, the circumferences of the waist and hips were measured to the closest 0.5 cm. The hip circumference was defined as the largest circumference across the buttocks and below the iliac crest, whereas the waist circumference (13) was defined as the point halfway between the iliac crest and the costal margins (lower ribs).

BMI: Weight in kilograms divided by height in meters squared yielded the body mass index (BMI). A BMI of 23.0–24.9 kg/m^2^ is considered overweight, but a BMI of 25 kg/m^2^ or higher is considered obese (14).

Hirsutism Scoring: The modified Ferriman-Gallwey score was used to quantify hirsutism and all subjects were evaluated for acne vulgaris, androgenic alopecia, and acanthosis nigricans (15).

#### 2.6.3 Biochemical investigation

Included liver function tests, kidney function tests, lipids, oral glucose tolerance test (OGTT) and 25-hydroxyvitamin D (25OHD) testing (done for selected cases only). Plasma glucose was measured using the glucose oxidase-peroxidase method.

#### 2.6.4 Hormonal investigation

Included test like thyroxine (T4), thyrotropin, luteinizing hormone (LH), follicle-stimulating hormone (FSH), prolactin (PRL), cortisol, anti-Müllerian hormone (AMH), dehydroepiandrosterone sulfate (DHEAS), 17-hydroxyprogesterone (17OHP), sex hormone binding globulin (SHBG), serum total testosterone, estradiol (E2), progesterone (P4) and insulin at 0, 30, and 120 min.

The blood samples for LH, FSH, testosterone, AMH, SHBG, and DHEAS were collected on day 3 to 7 (early follicular phase) of spontaneous menstrual cycle. Hormonal tests were analyzed using ECLIA technology on Cobas e411 analyzer (Roche Diagnostics USA).

#### 2.6.5 Radiological investigation

Patients were invited for ultrasound scan during follicular phase of their menstrual cycle. The scan was done in full bladder state which served as acoustic window for transabdominal assessment and empty bladder for transvaginal ultrasound. Uterine measurements are carried out by using high resolution ultrasonography machine.

Intra and extra observer variations: At each site, a single radiologist performed abdominal ultrasonography. Intra and extra observer variations between the radiologists were quantitated before and after a training session and minimized. Intra-class Correlation Coefficients (ICC) was used to assess inter-site reliability.

Probe Used: 2–5 MHz curvilinear probe (Mindray) was used for the transabdominal scan whereas 5–12 MHz probe was used for the transvaginal scan.

Uterine size: Linear measurements of length (craniocaudal), breadth (transverse) and height (Anteroposterior) on sagittal and transverse sections having maximum corresponding measurements was taken. For uterine length craniocaudal measurement extending from serosal surface of fundus to external cervical os was taken. For estimation of uterine width AP and Transverse measurements were taken as distance between corresponding serosal surfaces.

Uterine volume: Uterine volume estimation was done using the standard ellipsoid formula: *V* = 0.523 × L × W × H where V is uterine volume, L is uterine length, W is uterine width, H is anteroposterior height/thickness. The constant 0.523 is derived from the mathematical formula for the volume of an ellipsoid and is widely used in ultrasonographic volume estimation.

Endometrial thickness: The maximum thickness of endometrium perpendicular to long axis of uterine cavity in the sagittal scans with the complete endometrial lining visible all the way to the endocervical canal was taken to assess endometrial thickness (16). The echogenic area excluding the hypoechoic myometrium and fluid in the cavity was taken as the endometrial thickness.

Ovarian Volume: The ovarian volume estimate was calculated in ml by the formula for an ellipsoid, where D_1_, D_2_, and D_3_ are the three axial measurements: D_1_ × D_2_ × D_3_ × 0.52.

Ovarian Follicles: Follicles were counted during slow swiping movement of the transducer over the ovaries in two planes perpendicular to each other and taking care not to recount any follicle. Follicles measuring 2–9 mm in size only were included. The larger number was taken as follicle number per ovary (17). Data were statistically analyzed and Pearson’s correlation coefficient (r) was used to measure the strength of the association between two variables.

## 3 Results

Primarily, this was a study on the prevalence of PCOS, in which both cases and controls were recruited. The total sample of 1648 participants (control) comprised women who did not have any clinical or biochemical evidence of any disease, including PCOS. The baseline parameters of study population are represented in Table 1. Among the total women, 814 were from urban and 834 from rural areas. The mean age of women was 29.97 ± 6.44 years. The mean duration of shortest cycle is 28 days, and longest 34 days. The mean age of menarche was 13.07 ± 1.19. Urban–rural differences have been analyzed based on data from the current study. Among the urban women, the mean age of menarche was 13.00 ± 1.20 and that of rural women was 13.13 ± 1.19. The total number of menstrual cycles per year was 11.52 ± 1.32. For urban women the number was 11.54 ± 1.27. The total number of menstrual cycles per year among rural women was 11.49 ± 1.36.

**TABLE 1:** Baseline parameters of study population (*N* = 1648).

Baseline parameters		Mean ± SD	Median	IQR (Q_3_–Q_1_)
Age		29.97 ± 6.44	30	(25–35)
BMI		24.55 ± 4.23	24.06	(21.56–27.03)
Waist circumference		80.67 ± 12.15	79	(71.38–88.7)
Triceps thickness		19.78 ± 11.45	18	(12.07–26.3)
Length of menstrual cycle	Longest	33.44 ± 10.57	30	(28–33)
	Shortest	27.73 ± 6.49	27	(25–28)
Skin fold thickness		13.2 ± 16.87	2.5	(1.65–28.9)

The results of Intra-class Correlation Coefficients (ICC) between the radiologists show mixed levels of agreement: (Table 2)

**TABLE 2:** Intra-class Correlation Coefficients (ICC) for inter-site reliability of ultrasound measurements.

Type	ICC	F	df1	df2	*p*	Lower bound	Upper bound
ICC1	−0.1002	0.7267	8	18	0.6668	−0.33819243	0.3916
ICC2	0.1422	3.1697	8	16	0.0236	−0.03292863	0.5107
ICC3	0.4197	3.169	8	16	0.0236	0.0047727	0.7989
ICC1k	−0.3759	0.7267	8	18	0.6668	−3.13513554	0.6588
ICC2k	0.3322	3.1697	8	16	0.0236	−0.10575032	0.7579
ICC3k	0.6845	3.1697	8	16	0.0236	0.01418271	0.9226

1. Single-measure ICCs:
  a. ICC(1): −0.1002 (poor reliability)
  b. ICC(2): 0.142 (low reliability)
  c. ICC(3): 0.420 (moderate reliability)
2. Average-measure ICCs:
  a. ICC(1k): −0.376
  b. ICC(2k): 0.332
  c. ICC(3k): 0.684 (moderate-to-good reliability)

Notably, ICC (3k) = 0.684 (95% CI: 0.014–0.923, *p* < 0.05) indicates acceptable reliability under a consistency model. These findings suggest that while variability exists across sites, the standardized training contributed to achieving moderate agreement, particularly when averaging measurements.

### 3.1 Baseline parameters of study population

The mean BMI of the study subjects was 24.55 ± 4.23 kg/m^2^. They had a mean waist circumference of 80.67 ± 12.15 cm and triceps thickness of 19.78 ± 11.45 mm. The mean thickness of skin fold was 13.2 ± 16.87 mm, as represented in Table 1.

### 3.2 Normative sizes of female internal genital organs

(i) Normative sizes of female internal genital organs – transabdominal ultrasonography (TAS) (Tables 3, 4)

**TABLE 3:** Normative values of controls − TAS (*N* = 1233).

Parameters	Mean ± SD	2.5^th^ centile	97^th^ centile
Uterine length	7.09 ± 1.64	3.6	10.5
Uterine width	4.19 ± 1.02	2.3	6.38
Endometrial thickness	5.57 ± 2.65	0.5	10.7
Ovarian volume right	7.26 ± 3.29	2.1	14.1
Ovarian volume left	7.06 ± 3.44	1.88	14
Follicle number right	5.89 ± 3.03	1	13
Follicle number left	6.05 ± 3.18	1	13

**TABLE 4:** Normative value of controls − TAS (*N* = 1232).

Variable	Mean_SD	Median	IQR
Uterine volume	61.61 ± 37.24	55.58	(39.04–74.86)

The mean uterine length obtained with transabdominal scan was 7.09 ± 1.64 cm and uterine width 4.19 ± 1.02 cm. The estimated uterine volume with TAS in our study population was Mean ± SD: 61.61 ± 37.24 cm^3^, Median: 55.58 cm^3^, IQR: 39.04–74.86 cm^3^. The mean ovarian volume was 7.06 ± 3.44 cc on the left side and 7.26 ± 3.29 cc on the right side. There was a total of 6.05 ± 3.18 follicle number in the left and 5.89 ± 3.03 in the right. Normative parameters of transabdominal and transvaginal ultrasonogram are represented in Tables 2, 3 respectively.

(ii) Normative sizes of female internal genital organs – transvaginal ultrasonography (TVS) (Table 5)

**TABLE 5:** Normative value of controls − TVS (*N* = 740):

Parameters	Mean ± SD	2.5th centile	97th centile
Uterine length	7.11 ± 1.83	3.28	10.51
Uterine width	4.19 ± 1.19	2.1	6.8
Endometrial thickness	6.06 ± 2.39	2.3	11
Ovarian volume right	7.45 ± 3.28	2.03	13.74
Ovarian volume left	7.12 ± 3.38	2.01	14
Follicle number right	7.56 ± 4.35	2	19
Follicle number left	7.3 ± 4.12	2	18

Using transvaginal scan, the mean uterine length and width were respectively 7.11 ± 1.83 cm and 4.19 ± 1.19 cm. The population showed a mean endometrial thickness of 6.06 ± 2.39 mm. Ovarian volume of left and right were respectively 7.12 ± 3.38 cc and 7.45 ± 3.28 cc. The mean follicle numbers in right and left ovaries were 7.56 ± 4.35 and 7.3 ± 4.12.

### 3.3 Ovarian volume

(i) Correlation of various anthropometric parameters with ovarian volume (Table 6)

**TABLE 6:** Correlating ovarian volume with anthropometric, hormonal and metabolic parameters (*N* = 693).

Anthropometric parameters	Pearson r value	*P*-value
Age	−0.048	0.0933
Body weight	0.099***	0.0006
Height	0.184***	0
BMI	0.026	0.3589
Waist circumference	0.054	0.0615
Triceps thickness	−0.029	0.3255
FGS score	0.084**	0.0035
Skin fold thickness	−0.11***	0.0002
Hormonal parameters		
Prolactin	−0.006	0.8319
TSH	−0.017	0.571
T4	−0.005	0.8659
Testosterone	0.041	0.1736
DHEAS	0.024	0.4362
FSH	−0.046	0.1265
LH	−0.017	0.5692
SHBG	−0.022	0.457
17 OHP	−0.015	0.6551
CA 125	0.007	0.8261
Metabolic parameters		
LDL	0.025	0.3937
HDL	0.066*	0.0215
Triglycerides	0.077**	0.0079
Total cholesterol	−0.038	0.1892
Uric acid	0.032	0.2697

***Correlation is highly significant at 0.001.

**Correlation is significant at the 0.01.

*Correlation is significant at the 0.05.

The parameters of age showed negative correlation with ovarian volume with *P*-value 0.0933. However, body weight, height and Ferriman-Gallwey Score (FGS score) are positively correlated with ovarian volume with *p*-value of 0.0006, 0, and 0.0035, respectively.

(ii) Correlation of hormonal parameters with ovarian volume (Table 6):

Among the various hormones analyzed, testosterone showed significant positive correlation. LH and FSH showed negative correlation with ovarian volume with Pearson r value of −0.017 and −0.046 respectively.

(iii) Correlation of metabolic parameters with ovarian volume (Table 6):

Among the lipid parameters HDL, showed significant positive correlation with ovarian volume. The triglyceride levels showed an *r*-value of 0.077** with ovarian volume, which was statistically significant with a *p*-value < 0.01.

### 3.4 Endometrial thickness

(i) Correlation of various anthropometric parameters with endometrial thickness (Table 7):

**TABLE 7:** Correlating endometrial thickness with anthropometric and hormonal parameters (*N* = 725).

Anthropometric parameters	Pearson r value	*P*-value
Age	0.104***	0.0003
Body weight	−0.031	0.2732
Height	0.035	0.2185
BMI	−0.051	0.0766
Waist circumference	0.272***	0
Triceps thickness	−0.282***	0
FGS score	0.056*	0.0499
Skin fold thickness	−0.445***	0
Hormonal parameters		
Prolactin	0.013	0.6549
TSH	−0.129***	0
T4	0.048	0.1002
Testosterone	−0.002	0.9498
DHEAS	−0.017	0.5714
FSH	−0.065*	0.029
LH	−0.12***	0.0001
SHBG	−0.046	0.1675
17 OHP	0.107***	0.0007
CA125	0.104***	0.0003

***Correlation is highly significant at 0.001.

*Correlation is significant at the 0.05.

Among the parameters analyzed age, height, waist circumference and Modified Ferriman Gallaway (FGS) score showed positive correlation with endometrial thickness. Age has a *r*-value of 0.104***. Waist circumference is significantly associated with endometrial thickness has *r*-value of 0.272*** and *p*-value of <0.001. FGS score has *r*-value of 0.056* and *p*-value of 0.0499. Skin fold thickness, triceps thickness and BMI showed negative correlation with endometrial thickness which was statistically significant with *p*-value < 0.001.

(ii) Correlation of various hormonal parameters with endometrial thickness (Table 7):

Among the hormones analyzed, the ones which showed significant positive correlation with endometrial thickness were OHP (hydroxyprogesterone), CA 125. The Pearson *r*-value of CA 125 when correlated with ovarian volume was 0.104***, with a statistical significance of *p* < 0.01. But SHBG levels showed negative correlation with endometrial thickness. FSH showed a *r*-value of −0.065* and *p*-value of 0.029. TSH showed an *r*-value of −0.129*** and (LH −0.12***), both were statistically significant with *p*-value < 0.001.

## 4 Data analysis

The analysis was performed using SPSS version 16.0, generating descriptive statistics, normative values, and correlation results for anthropometric, hormonal, and metabolic parameters. The findings provide clear insights into baseline characteristics and their associations with ovarian and endometrial measurements. Significant correlations were identified at both the 0.01 and 0.05 levels, highlighting key physiological relationships within the study population. Overall, the SPSS output offers a comprehensive statistical overview supporting further interpretation.

Besides the median, reference intervals were determined at 2.5th and 97.5th percentiles. Mean, median and standard deviations were computed for each of the baseline and radiological parameters of the study participants. Moreover, literature survey for comparative analysis was also performed for comparing the data from the present study with the already published data.

## 5 Discussion

This multicentric study was primarily designed to establish normative data on ultrasonographic parameters among clinically and biochemically healthy women, while also exploring urban–rural variations. It is well recognized that reference ranges may vary across different racial and ethnic groups. Although normative values of female internal genital organs have been extensively validated in western populations, similar data are limited for South Asian women. Therefore, the present study aimed to generate population-specific reference values for ultrasonographic parameters in healthy Indian women. To the best of our knowledge, this is the first large pan India study to report sonographic normative range of uterine and ovarian dimensions. The inclusion of 1648 clinically and biochemically normal women from nine geographically diverse sites provides a comprehensive representation of baseline characteristics among Indian women of reproductive age. The mean age of participants was approximately 30 years, with a balanced distribution between urban and rural populations.

The uterine volume, ovarian volume, follicle count, and endometrial features are the key indicators in ultrasonography used to evaluate women’s reproductive health. The measurement of ovarian volume aids in the diagnosis and management of various puberty and adult reproductive abnormalities. It also serves as an indirect predictor of the ovarian reserve in women of childbearing age. The measurement of ovarian volume is an indirect indicator of the ovarian reserve in women of reproductive age, and it helps in the identification and treatment of several disorders of adolescence and adult reproductive function. The measurement of uterine volume enhances the applicability of the findings, particularly for clinical assessment and surgical planning.

Growth hormone also has a role in measuring the volume and growth of the ovaries during childhood and adolescence. This concept was suggested by data from Bridges et al. in 1993, who investigated girls with growth disorders involving GH insufficiency, skeletal dysplasia, and tall stature (18).

Compared to GH-insufficient females receiving GH treatment the total ovarian volume of untreated GH-insufficient girls was substantially lower. Additionally, they discovered that tall girls’ ovarian volumes were noticeably larger than those of either of the GH-treated groups.

Due to insufficient data density across all age intervals (18–40 years), statistically stable nomogram curves could not be generated reliably; and future studies with larger datasets may address this aspect.

### 5.1 Ovarian volume

Currently, one of the diagnostic criteria for polycystic ovary syndrome which is the most prevalent endocrinopathy in women is ovarian volume. Ovarian volume is also of value in screening for ovarian cancer. Young girls who experience early puberty have noticeably larger ovarian volume (19).

Hence its measurement is useful during the workup of precocious puberty. Ovarian volume is a useful differentiator between central precocious puberty and premature thelarche. Furthermore, measurement of ovarian volume is a useful indicator to assess the efficacy of treatment of central precocious puberty with GnRH analogues.

Kelsey et al. reported the first validated normative model of ovarian volume from conception to old age, establishing reference values across the female lifespan that may aid in the diagnosis and management of a wide range of gynecological and reproductive disorders (20). Ovarian volume increases through childhood and adolescence and reaches its peak at 20 years of age, thereafter declining toward the menopause and beyond. Their study model shows that in the average case ovarian volume rises from 0.7 mL (95% CI 0.4–1.1 mL) at 2 years of age to a peak of 7.7 mL (95% CI 6.5–9.2 mL) at 20 years of age and further declines throughout life to about 2.8 mL (95% CI 2.7–2.9 mL) at the menopause. In the current study also, ovarian volume was shown to have negative correlation with age.

The upper limit of ovarian volume was shown to be around 14 cc in the current study. The Rotterdam criteria for PCOS suggest a cut off 10cc of ovarian volume for diagnosis. Thus, the study results may suggest a higher cut off to be considered among Indian women.

In the current study, ovarian volume shows significant positive correlation with HOMA IR and testosterone. The generation of excess androgen by the ovarian theca and stroma has been directly associated with elevated insulin levels (21). These results were consistent with other study by Handelsman et al. where ovarian volume of >10 mL was associated with a higher BMI, waist circumference, and 60-min post glucose load insulin levels (22).

### 5.2 Uterine dimensions

The knowledge of normal uterine dimensions, its ranges and its correlation with varying clinical conditions is essential in predicting various pelvic organ pathology. In the current study, the mean uterine length obtained with transabdominal scan (TAS) was 7.09 ± 1.64 cm and uterine width obtained was 4.19 ± 1.02 cm. The estimated uterine volume with TAS in our study population was 61.61 ± 37.24 cm^3^. While using transvaginal scan, the mean uterine length and width were respectively 7.11 ± 1.83 cm and 4.19 ± 1.19 cm. The population showed a mean endometrial thickness of 6.06 ± 2.39 mm.

In parous women, as age increases from 21 to 40, the uterine length increases, and it tends to decrease from 41 to 60 years age. Increases in body height, weight, and surface area all result in an increase in uterine length. In women who are parous, there is a positive correlation of uterine length with age, body weight, height, and surface area.

In a previous study by Ameer et al., adult female uterine dimensions found was 8 cm long, 5 cm across, and 4 cm anteroposterior and an average volume of 80–200 mL (23). Becker and colleagues reported the normal ranges obtained from transvaginal scan in uterine dimensions and calculated uterine weight, based on examination of 839 patients with normal uteruses and of 85 patients before planned hysterectomy (24). The difference in uterine size (including all three measured parameters) between premenopausal women with one or more deliveries and nulliparous women was consistent. The statistical mean of uterine length obtained in the group of multiparas was 9.2 cm ± 0.8 compared to 7.3 cm ± 0.8 of nulliparas (25). A study by Merz et al. (26) showed uterine dimensions in nulliparous women to be 4.0 cm ± 0.6 cm, 7.3 cm ± 0.8 cm, and 3.2 cm ± 0.5 cm for AP, longitudinal, and transverse dimensions, respectively.

Broekmans et al. (27) showed the uterine dimensions for nulliparous women to be 7.0, 4.0, and 4.0 cm for length, width, and AP dimensions, respectively. Another study was done by Parmer et al. (17) in 80 women (40 parous and 40 nulliparous) determined the normal range of the uterine dimensions and correlated with body height, body weight, and body surface area in parous and nulliparous women. The average size of uterus found in parous women was 9.07 × 5.19 × 4.14 cm and in nulliparous women was 7.10 × 4.52 × 3.27 cm. Tassoni et al. (28) did a metanalysis of a total of 33 studies and concluded that length of normal uterus is about 50–80 mm in fertile age. The cutoff value for endometrial thickness in postmenopausal women who are asymptomatic is up for debate, however a measurement of >5 mm may indicate postmenopausal hemorrhage, which calls for more testing.

### 5.3 Ovarian follicle

Ovarian follicle number is another useful parameter from pelvic ultrasonogram. Ovarian antral follicle count (AFC) is recognized as a reliable surrogate marker. It helps with assessment of infertility, assisted reproduction, menopause risk prediction and diagnosis of hyperandrogenic anovulation. The most feasible approach for AFC estimation in clinical practice is to count all recognizable follicles with a diameter of 2–9 mm, eliminating the need to measure multiple follicles (27).

A key measure of ovarian reserve and reproductive potential is the number of follicles in the ovary, often known as the follicular number per ovary (FNPO). Compared to only reporting the total follicle count across both ovaries, evaluating the FNPO offers more practical and informative insight in gynecological clinical practice. Clinicians can use this metric to monitor ovarian response during assisted reproductive treatments, examine general ovarian health, and evaluate disorders including polycystic ovarian syndrome (PCOS) (29).

Antral follicle count declines with chronologic age in women. AFC < 5–7 is associated with small number of oocytes retrieved (30) and reduced pregnancy rate. In the current study, average follicle count (AFC) is around 6 by transabdominal and 7 by transvaginal scan. An increased risk of ovarian hyperstimulation syndrome and an excessive ovarian response are associated with a total AFC ≥ 20 (31).

A study on age-specific nomogram for the decline in antral follicle count throughout the reproductive period was done by Marca et al. (32) in 115 premenopausal women. The analysis showed AFC declines linearly with age; the median AFC drops by 0.4 for each additional year of age.

## 6 Limitation

1. Considering the vast and diverse Indian population, the sample size of 1,648 participants may be insufficient to generalize the findings to all Indian women.
2. The inclusion of both nulliparous and parous women resulted in a mixed population; selecting one specific group would have allowed the results to be more representative of that population. Parity-specific information was not recorded in our original data collection sheet. We recommend that future studies incorporate parity as a key variable to enable more detailed subgroup analyses.
3. Despite being conducted across 9 sites, this cross-sectional study included only women who were clinically and biochemically normal, thereby limiting the generalizability of the findings to women with metabolic or reproductive disorders.
4. The cross-sectional design and no longitudinal follow-up restrict the ability to establish causal relationships between ovarian morphology with hormonal profiles, and metabolic parameters.
5. Potential confounding factors such as lifestyle, parity, and socioeconomic status were not fully adjusted.
6. Certain anthropometric measurements, such as skinfold thickness, are prone to inter-observer variation, and urban–rural differences were not explored in depth.

Therefore, in the future, a well-designed longitudinal cohort study focusing specifically on either parous or nulliparous women would be valuable. Such a study would enable the assessment of changes in ovarian morphology, hormonal dynamics, and metabolic profiles over time, thereby helping to establish causal relationships among these parameters. Longitudinal data would also facilitate the identification of normal physiological variations across different age groups and reproductive stages, allowing for the development of more accurate, population-specific normative ranges.

Such evidence would enhance the clinical interpretation of ultrasonographic and biochemical findings in Indian women and guide more precise diagnostic and screening criteria for reproductive and metabolic health.

## 7 Conclusion

The normative range of ovarian volume, follicle number, and endometrial thickness during the proliferative phase of the menstrual cycle has been established by this extensive community-based survey of reproductive-age women from 6 different regions of India. Age and ovarian volume have negative correlation. While height, hirsutism score, insulin resistance, and serum androgen levels are positively correlated with ovarian volume. Endometrial thickness has a negative correlation with triceps skin fold thickness and a positive correlation with age, waist circumference, and hirsutism score.

## Data Availability

The raw data supporting the conclusions of this article will be made available by the authors, without undue reservation.
